# Pharmacy Technicians’ Willingness to Perform Emerging Tasks in Community Practice

**DOI:** 10.3390/pharmacy6040113

**Published:** 2018-10-12

**Authors:** William R. Doucette, Jon C. Schommer

**Affiliations:** 1 College of Pharmacy, University of Iowa, 115 S. Grand Avenue, S518 PHAR, Iowa City, IA 52242, USA; 2 College of Pharmacy, University of Minnesota, 308 Harvard Street, S.E., Minneapolis, MN 55455, USA; schom010@umn.edu

**Keywords:** pharmacy technician, community pharmacy, willingness, services

## Abstract

New tasks are being developed for pharmacy technicians in community practice. The objectives of this study were to (1) assess the willingness of community pharmacy technicians to perform new tasks, and (2) to identify factors affecting technicians in assuming new tasks in community pharmacy practice. An online survey asked about the respondent characteristics, involvement in pharmacy technician tasks, willingness to perform emerging pharmacy technician tasks, and influences on pharmacy technicians’ performance of emerging tasks. Descriptive statistics were calculated for all items. A total of 639 usable surveys from community pharmacy technicians were used in the analyses. The respondents reported a mean of 11.5 years working as a pharmacy technician, with 79.2% working full time. Technicians reported high willingness to perform four emerging tasks, moderate willingness for six tasks, and low willingness to perform two tasks. The low willingness tasks were administering a vaccination and drawing a blood sample with a finger stick. Four workplace influences on willingness to perform emerging tasks were insufficient staffing, insufficient time to complete additional tasks, employers not classifying technicians based on specialized skills, and usually feeling stress at work. It appears likely that pharmacy technicians will be willing to perform the new tasks needed to support the emerging patient care services in community pharmacies.

## 1. Introduction

Community pharmacy practice continues to evolve as payers seek higher quality care for limited payment, providers pursue improved coordination of care, healthcare systems strive for collaborative care models, and chronically ill patients prefer care from a pharmacist they know [1,2,3]. As new community pharmacy services are expanded and developed, typically there are changes in workflow and staffing within the pharmacies. While pharmacists are being called upon to provide greater patient care through collaborative and other innovative healthcare models, pharmacy technicians are also undertaking new roles in community pharmacy practice. New roles for pharmacy technicians are derived from new services, such as medication management [4,5], technician prescription validation (i.e., tech-check-tech) [6], medication reconciliation [7], medication synchronization [8], and immunizations [9,10,11]. Although some growth has occurred with these services, as pharmacy payment and care models also evolve, it is likely that these services, and others, will continue to become a part of community pharmacy practice [12,13]. To succeed in this environment, community pharmacies will need a strong supply of well-trained and motivated pharmacy technicians. Such technicians can free up pharmacists to deliver additional patient care services, while performing new tasks themselves. The purpose of this study was to evaluate the willingness of pharmacy technicians to perform new tasks as community pharmacy practice changes in the future.

The specific objectives of this study were as follows:

(1) Assess the willingness of pharmacy technicians to perform new tasks in community pharmacy practice;
(2) Identify factors that can affect pharmacy technicians in assuming new roles in community pharmacy practice.

## 2. Materials and Methods

This study was an online survey sent to 29,084 certified pharmacy technicians. The sample was obtained through the Pharmacy Technician Certification Board (PTCB) and the National Health-Career Association (NHA). The sample frames did not support a priori screening to identify technicians working in community pharmacies, so such screening was done in the survey itself. A Qualtrics online survey was prepared by the research team, and links to it were sent out via email by PTCB and NHA. Three email contacts containing a survey link were sent to the sample about 10 days apart from each other. The survey was open for 40 days overall.

The survey asked about four primary topics: respondent characteristics, involvement in pharmacy technician tasks, willingness to perform emerging pharmacy technician tasks, and potential influences on pharmacy technicians’ performance of emerging tasks. See the supplementary material for the formatted survey questions. The respondent characteristics included several demographics (e.g., age, gender), as well as practice-related variables (e.g., practice experience). Technician involvement in 12 emerging pharmacy technician tasks was rated with a 3-point scale (1 = not at all involved, 2 = somewhat involved, 3 = regularly involved). Respondents also rated their willingness to perform the 12 emerging pharmacy technician tasks using a 4-point scale (1 = unwilling, 2 = slightly willing, 3 = moderately willing, 4 = very willing). Finally, the survey respondents used a 7-point Likert scale (1 = strongly disagree, 2 = disagree, 3 = somewhat disagree, 4 = neither agree nor disagree, 5 = somewhat agree, 6 = agree, 7 = strongly agree) to rate workplace variables. The list of emerging technician tasks was developed from the literature and with input from community pharmacy informants. Based on the willingness ratings of respondents, the emerging tasks were categorized as high willingness (at least 60% very willing), moderate willingness (25–59% very willing) and low willingness (less than 25% very willing). Descriptive statistics (e.g., mean, median, frequencies) were calculated for all items, using data from respondents who reported current employment as a pharmacy technician in a community pharmacy setting. This study was approved by the University of Iowa Human Subjects Office.

## 3. Results

A total of 1257 survey responses were received. Of these, 639 were usable surveys from people working as community pharmacy technicians which were used in the analyses described in this article. The overall response rate was 4.3 percent. All of these respondents reported being a certified pharmacy technician. The most common setting type was large chain community pharmacy (36.2%), followed by mass merchandiser (28.8%), independent (15.2%), supermarket (10.6%) and other (9.3%). The regions the respondents came from were the Midwest (39.1%), South (37.6%), West (15.4%) and Northeast (7.9%). The respondents reported a mean of 11.5 (S.D. = 8.7) years working as a pharmacy technician. A majority (79.2%) of respondents reported full-time employment (>30 h a week). Similarly, a large percentage (87.5%) of respondents were female. The mean age of the community pharmacy technician respondents was 42.5 (S.D. = 12.4) years. Over half (54.3%) of the respondents reported their primary method of training to work as a pharmacy technician was unaccredited on-the-job training from their employer. Other reported training methods included unaccredited structured training program from their employer (11.3%), accredited standalone training program (10.6%), accredited structured training program from their employer (10.2%) and other (13.5%).

At least half of the community pharmacy technician respondents reported regular involvement with only two of the emerging tasks (Table 1): calling a prescriber for clarification of a prescription order (56.2%) and collecting patient medication history from a patient (51.3%). Less than 20% of respondents reported regular involvement with eight of the emerging tasks. This last set of activities has historically been performed by pharmacists, often due to legal restrictions preventing technicians from performing them. Three of these activities are related to dispensing: performing final prescription verification, taking a prescription order over the telephone, and transferring a prescription to another pharmacy. The three least commonly performed tasks were part of relatively new services, and perhaps were beyond the traditional scope of pharmacy technician roles: obtaining patient vital signs, administering a vaccination, and drawing a blood sample with a finger stick.

The respondents also reported their willingness to perform the 12 emerging technician tasks. For four of these tasks, at least 60% of the respondents stated that they would be very willing to perform them, while less than 8% said they were unwilling to do them (Table 1). These “high willingness” tasks were calling a prescriber to clarify a prescription order, collecting patient medication history, documenting patient care, and calling patients prior to medication synchronization filling. Another set of six tasks had lesser willingness, being rated as very willing to perform by 37–53% of respondents, and showing greater unwillingness (17–29%).

Two final “low willingness” tasks were rated as very willing to perform by less than 25% of respondents, and unwilling to perform by at least 47% of respondents: administering a vaccination, and drawing a blood sample with a finger stick.

The final section of the survey asked respondents about 11 workplace variables that could affect their willingness to perform emerging pharmacy technician tasks. For reporting, the number and percentage of respondents who rated any level of agreement (i.e., somewhat agree, agree, strongly agree) were calculated for each variable, as well as the scale mean and standard deviation. Seven variables expected to support pharmacy technician willingness to perform emerging tasks had agreement from at least 59% of respondents (Table 2). The remaining four variables showed less likelihood to support technicians’ performance of new tasks, having strong agreement with a negative factor (1 item) or having low agreement with a positive factor (3 items). These four variables were that current staffing may not support technicians assuming new tasks (negative item), having enough time to complete additional tasks, employers classifying technicians based on specialized skills, and not usually feeling stress at work.

## 4. Discussion

There were four emerging tasks with moderate involvement ratings. Three of these moderate involvement tasks were related to communication: two with patients (i.e., collecting patient medication history and calling prior to medication synchronization filling) and one with providers (i.e., calling a prescriber for clarification of a prescription order). These tasks show the importance of communication skills in expanding the roles of community pharmacy technicians. Technicians that regularly interact with patients need to be effective in gathering information from them, as well as in giving pertinent details to them. The other task categorized in the moderate involvement sector was documenting patient care. This task would typically be outside of dispensing, for which documentation occurs automatically through the dispensing software. Rather, this task likely relates to non-dispensing services, such as documentation of immunizations in state registries and of medication management services. Lengel et al. reported low participation by trained pharmacy technicians in documenting medication therapy management (MTM) services [5].

Based on reported technician involvement, eight tasks were categorized as low involvement. These tasks may not be performed by technicians in many community pharmacies due to legal or regulatory restrictions. For example, many states do not currently allow technicians to perform the final prescription verification in community pharmacies. Desselle and Holmes also reported technician verification of other technicians’ work as having low involvement from community pharmacy technicians [14]. Similarly, transferring a prescription to another pharmacy and taking a prescription order over the telephone are restricted to pharmacists in some states. Until state laws address these tasks, it is likely they will not be shifted largely to technicians. Conducting medication reconciliation after discharge from a hospital was reported as being done regularly by almost 10% of respondents in this study. Given the strong interest in smooth care transitions, this could be a task that becomes more common as more community pharmacies deliver such a service. Three tasks had no more than 2% of respondents rate that they are regularly involved in them: obtaining patient vital signs, administering a vaccination, and drawing a blood sample with a finger stick. These tasks are not widely performed in community pharmacies by technicians. Thus, it is likely that community pharmacy technicians have little or no opportunity to perform these tasks at this time. Desselle et al. also reported very low involvement by pharmacy technicians in administering immunizations [15].

The respondents’ involvement ratings indicate that most of these emerging tasks are not widespread in community pharmacy practice. Considering the involvement and willingness to perform ratings together, it can be seen that respondents tended to be less willing to perform tasks with which they had lower involvement. That is, some of the unwillingness to perform might simply derive from a lack of familiarity with the low-rated tasks. Another characteristic of the two tasks with the lowest willingness ratings is they involve needles. It could be that some of the respondents are not comfortable with such activities, which may be outside of their technician training.

Over half (54.3%) of respondents stated they used unaccredited on-the-job training from their employer, while the next most frequent method was unaccredited structured training program from their employer (11.3%). In addition, another 10.2 percent of respondents reported using an accredited structured training program from their employer. Thus, about three-fourths (75.8%) of the respondents completed technician training through their employer. Accredited technician training programs accounted for just over 20%, between accredited standalone (10.6%) and accredited employer (10.2%) programs. It is clear that employer-led training programs are currently, and will likely continue to be, a key factor in training pharmacy technicians for emerging roles. As new standards are developed for training pharmacy technicians, their employers are likely to influence the future content and delivery of such training.

Based on technician ratings, seven of the work environment variables very likely would support new technician tasks, while four would be less supportive or even unsupportive. Four of the more supportive workplace characteristics represented organizational factors: technicians working well with pharmacists, managerial support for technicians performing new tasks, technicians receiving training to perform new tasks, and support of fellow pharmacy staff to implement a new service. As new activities are introduced into a workplace, the support of the employees at all levels is needed for the activities to be performed properly. These findings point to the need for technicians to be supported by their managers and fellow pharmacy staff when taking on new pharmacy technician duties. The other three more supportive variables were characteristics of individuals: confidence to learn new tasks, belief that new tasks keep work interesting, and being excited about implementing a new service. These individual characteristics could be considered at the time of hiring technicians. In addition, confidence relates to self-efficacy, which can be increased by education, mastery experience and social modeling [16]. 

The four workplace characteristics that were expected to be less supportive of technicians performing new tasks included current staffing being unable to support new tasks, insufficient time in the current workday to support new tasks, feeling stress at work, and the employer not classifying technicians based on specialized skills. Any workplace that introduces new tasks will need to be aware of how the new tasks affect employee time and workload. Pharmacies that utilize technicians in new roles to implement new services will need to manage the workload of their technicians. It is expected that new tasks would add to technicians’ stress level, which likely could be managed by training and experience. As technicians begin to perform new tasks, some of them will need specialized skills and training. Training and some type of credentialing could be used to support technicians obtaining advanced knowledge and skills.

This study has several limitations. The low response rate limits its generalizability to the population of community pharmacy technicians. Thus, the findings should be interpreted conservatively. Another limitation is that many of the questions had not been used in previous studies. To try to assure clear understanding by respondents, the items were evaluated and improved through a cognitive testing process in which pharmacy technicians read the items aloud and provided feedback on vague language. Also, content experts read the survey and provided feedback prior to the main survey. It is possible that pharmacy technicians’ willingness to perform emerging tasks varies by state due to differences in state law. Although this was a national sample, over half of the states had less than ten responses. Future research could be done to examine the effects of state laws and regulations on technicians’ willingness to perform new tasks. Finally, the limited detail collected in the survey about technician training did not allow study of the extent to which these emerging topics are being taught during technician training. Again, future research is recommended to investigate this issue. 

## 5. Conclusions

Overall, community pharmacy technicians reported being willing to perform almost all of the emerging tasks that were rated in this study. It is likely that technicians will be able to increase their roles where community pharmacy practice offers expanded and new patient care services and where permitted by law. As pharmacies work to integrate into collaborative care and other innovative care models, it appears likely that pharmacy technicians will be willing to perform the new tasks needed to support the evolution and advancement of patient care services delivered in the community pharmacy setting.

## Figures and Tables

**Table 1:** Involvement ^A^ and willingness ^B^ to perform emerging technician tasks.

Emerging Technician Tasks	Not at All Involved n, (%)	Regularly Involved n, (%)	Unwilling n, (%)	Very Willing n, (%)
Call prescriber for clarification of electronic or written prescription order	95, (14.9)	359, (56.2)	34, (5.3)	482, (75.4)
Collect patient medication history from patient	111, (17.4)	328, (51.3)	40, (6.3)	418, (65.4)
Document pharmacy care in patient records	220, (34.4)	204, (31.9)	41, (6.4)	403, (63.1)
Call patient prior to medication synchronization to clarify medications to be filled	188, (29.4)	236, (36.9)	49, (7.7)	389, (60.9)
Prepare vaccination for administration by pharmacist	392, (61.3)	121, (18.9)	144, (22.5)	337, (52.7)
Take prescription order from physician over the telephone	457, (71.5)	90, (14.1)	137, (21.4)	320, (50.1)
Transfer a prescription to another pharmacy	437, (68.4)	71, (11.1)	117, (18.3)	314, (49.1)
Conduct medication reconciliation after patient is discharged from hospital	443, (69.3)	58, (9.1)	111, (17.4)	284, (44.4)
Obtain patient vital signs (blood pressure, heart rate, temperature)	594, (93.0)	13, (2.0)	186, (29.1)	279, (43.7)
Perform final prescription verification during dispensing	423, (66.2)	125, (19.6)	157, (24.6)	240, (37.6)
Administer a vaccination to a patient	599, (93.7)	11, (1.7)	301, (47.1)	156, (24.4)
Draw a blood sample from a patient (finger stick)	617, (96.6)	6, (0.9)	311, (48.7)	151, (23.6)

^A^ Involvement rated with a 3-point scale: 1 = not at all involved, 2 = somewhat involved, 3 = regularly involved. ^B^ Willingness rated with a 4-point scale: 1 = unwilling, 2 = slightly willing, 3 = moderately willing, 4 = very willing.

**Table 2:** Work environment variables.

Rate your level of agreement with each of the following statements in regard to pharmacy technicians working in your pharmacy.	Agreement n, (%)	Mean (SD)
At my workplace the technicians work well with the pharmacists.	583 (91.5%)	6.12 (1.22)
If I had to learn to do new tasks for my job, I feel confident that I could do so readily.	580 (91.2%)	6.14 (1.16)
Performing new tasks in my workplace helps keep my work interesting.	552 (86.7%)	5.91 (1.33)
I would be excited to have a new opportunity if we implemented a new service at my pharmacy.	515 (80.8%)	5.69 (1.51)
At my workplace the managers would be supportive when technicians are performing new tasks.	472 (74.1%)	5.31 (1.57)
When implementing a new service, technicians at my workplace may receive training to perform the tasks.	457 (71.7%)	5.05 (1.66)
Current staffing levels at my pharmacy may not support technicians assuming additional tasks.	427 (67.0%)	4.98 (1.85)
My fellow pharmacy staff would be supportive of implementing a new service in the pharmacy.	375 (59.0%)	4.78 (1.67)
There is enough time in my current workday to complete additional tasks and responsibilities.	233 (36.6%)	3.43 (2.01)
My employer classifies technicians based on specialized skills.	225 (35.4%)	3.78 (1.89)
I don’t usually feel stress when working at my job.	224 (35.2%)	3.43 (1.97)

Agreement was rated on a 7-point scale: 1 = strongly disagree, 2 = disagree, 3 = somewhat disagree, 4 = neither agree nor disagree, 5 = somewhat agree, 6 = agree, 7 = strongly agree. Agreement variable = sum of somewhat agree, agree and strongly agree. Note: Unshaded variables are expected to support technician willingness to perform new tasks, while shaded variables are not.

## Supplementary Materials

The following are available online at http://www.mdpi.com/2226-4787/6/4/113/s1, Formatted Survey Questions.

## References

[1] Easter J.C., DeWalt D.A. (2017). The medication optimization value proposition: Aligning teams and education to improve care. NCJM.

[2] Trygstad T. (2017). Payment reform meets pharmacy practice and education transformation. NCJM.

[3] Urick B.Y., Prown P., Easter J.C. (2017). Achieving better quality and lower costs in Medicaid through enhanced pharmacy services. NCJM.

[4] Powers M.F., Bright D.R. (2008). Pharmacy technicians and medication therapy management. J. Pharm. Technol..

[5] Lengel M., Kuhn C.H., Worley M., Wehr A.M., McAuley J.W. (2018). Pharmacy technician involvement in community pharmacy medication therapy management. J. Am. Pharm. Assoc..

[6] Adams A.J., martin S.J., Stolpe S.F. (2011). “Tech-check-tech”: A review of the evidence on its safety and benefits. Am. J. Health-Syst. Pharm..

[7] Kraus S.K., Sen S., Murphy M., Pontiggia L. (2017). Impact of a pharmacy technician-centered medication reconciliation program on medication discrepancies and implementation of recommendations. Pharm. Pract..

[8] America’s Pharmacist (2013). All Aboard the Synchronization Train.

[9] Powers M.F., Hohmeier K. (2011). Pharmacy technicians and immunizations. J. Pharm. Technol..

[10] Bright D., Adams A.J. (2017). Pharmacy Technician-Administered Vaccines in Idaho (letter). Am. J. Health-Syst. Pharm..

[11] McKeirnan K.C., Frazier K.R., Nguyen M., MacLean L.G. (2018). Training pharmacy technicians to administer immunizations. J. Am. Pharm. Assoc..

[12] Bryan K., Mattingly T.J.I.I., Gernant S.A. (2018). Optimizing the Role of Pharmacy Technicians in Patient Care Settings. J. Am. Pharm. Assoc..

[13] Mattingly A.N., Mattingly T.J. (2018). Advancing the Role of the Pharmacy Technician: A Systematic Review. J. Am. Pharm. Assoc..

[14] Desselle S.P., Holmes E.R. (2017). Results of the 2015 National Certified Pharmacy Technician Workforce Survey. Am. J. Health-Syst. Pharm..

[15] Desselle S.P., Hoh R., Holmes E.R., Gill A., Zamora L. (2018). Pharmacy technician self-efficacies: Insight to aid future education, staff development, and workforce planning. Res. Soc. Adm. Pharm..

[16] Bandura A. (2004). Health promotion by social cognitive means. Health Educ. Behav..

