# Supplementary material for: Pharmacy Technicians’ Willingness to Perform Emerging Tasks in Community Practice
Source: Pharmacy (Basel). 2018 Oct 12;6(4):113. doi: 10.3390/pharmacy6040113 (PMC6306904; doi:10.3390/pharmacy6040113)
Supplement: Supplementary file 1 [file pharmacy-06-00113-s001.zip › pharmacy-359478-supplementary.pdf]

Formatted Survey Questions

2017 National Pharmacy Technicians Survey on Emerging Tasks

Start of Block: Intro

Intro

Welcome to the 2017 National Pharmacy Technicians Survey on Emerging Tasks. We value your professional opinion and would appreciate it if you would take a few minutes to complete this short survey. Please click the NEXT button to take the survey.

End of Block: Intro

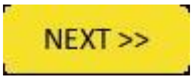

Start of Block: Block1-Screening [response Q1(1) continues; Q1(2) – Q1(6) goes to Q2-S]

Q1 Please check the response that best matches your current employment status.

- Currently employed as a pharmacy technician. (1)
- Employed in a health or pharmacy-related field or position, but not functioning as a pharmacy technician. (2)
- Retired. (3)
- Unemployed - Seeking employment. (4)
- Unemployed - Not seeking employment. (5)
- Other - Please specify: (6) ____

End of Block: Block1-Screening

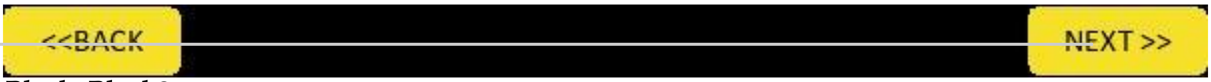

Start of Block: Block2

Q2 Are you currently certified as a pharmacy technician?

- o Yes (1)
- o No (0)

Q3 Please check the ONE item that best describes your primary place of employment.

[Q3(1) - Q3(6) are community-based.]

- o Independent community pharmacy (fewer than 4 stores under same ownership) (1)
- o Large chain community pharmacy (>50 units under same ownership, e.g., CVS, Walgreens) (3)
- o Supermarket pharmacy (5)
- o Government hospital/health system - INPATIENT (7)
- o Non-government hospital/health system - INPATIENT (9)
- o Specialty drug pharmacy (11)
- o Nursing home/Long-term care (13)
- o Ambulatory care (e.g., medical clinic, office-based practice, non-dispensing pharmacy) (15)
- o Pharmaceutical industry (17)
- o Small chain community pharmacy (4 to 50 stores under the same ownership) (2)
- o Mass merchandiser, e.g., Costco, Target, Wal-Mart) (4)
- o Clinic-based pharmacy (licensed pharmacy located in or near a clinic) (6)
- o Government hospital/health system - OUTPATIENT (8)
- o Non-government hospital/health system - OUTPATIENT (10)
- o Pharmacy benefit administration (e.g., PBM, managed care) (12)
- o Mail service pharmacy (14)
- o Pharmacy technician training program (e.g., vocational school, community college) (16)
- o Other organization - please specify: (18)

____

[No BACK Button on this page is intentional]

End of Block: Block2

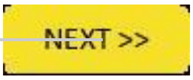

Start of Block: Block3

Q4 In which state is your primary employment? (Please select from the drop down list.)

▼ AL, Alabama (1) ... WY, Wyoming (50)

Q5 What is the population of the community in which your primary employment is located?

- o Less than 10,000 (Rural/Small Community) (1)
- o 10,000 - 49,999 (Small Metro) (2)
- o 50,000 - 99,999 (Metro) (3)
- o 100,000 or more (Large Metro) (4)

Q6 How many years have you worked as a pharmacy technician? (round to nearest whole number)

________ years (1)

Q7 How many years have you worked for your current employer?

________ years (1)

Q8 Do you work full time or part time?

- o Full time (greater than 30 hours per week) (3)
- o Part time (20 - 30 hours per week) (2)
- o Part time (less than 20 hours per week) (1)

Q9 Indicate which of the following best describes your PRIMARY method of training to work as a pharmacy technician?

- ○ On-the-job training from my employer, unaccredited (1)
- ○ Self-guided training using books and online resources (2)
- ○ Structured training program from my employer, unaccredited (3)
- ○ Structured training program from my employer, ASHP/ACPE accredited (4)
- ○ Standalone training program (e.g. vocational school, community college), unaccredited (5)
- ○ Standalone training program (e.g. vocational school, community college), ASHP/ACPE accredited (6)

End of Block: Block3

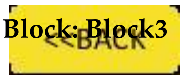

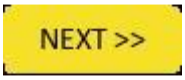

Start of Block: Block4

Q10 Please indicate your level of involvement for each activity listed below during a typical work week.

	Not at all Involved (1)	Somewhat Involved (2)	Regularly Involved (3)
Receive prescription orders (Q10_1)	○	○	○
Collect and communicate patient specific information (Q10_2)	○	○	○
Assess prescription for completeness, accuracy, authenticity, legality and reimbursement eligibility (Q10_3)	○	○	○
Process a prescription order (Q10_4)	○	○	○
Provide medication to patient/patient's representative (Q10_5)	○	○	○
Direct patient to pharmacist for counseling (Q10_6)	○	○	○
Identify pharmaceuticals and supplies to be ordered and manage inventory (Q10_7)	○	○	○
Use and maintain automated and point-of-care dispensing technology (Q10_8)	○	○	○
Communicate with third party payers to determine coverage for products and services (Q10_9)	○	○	○
Perform final prescription verification during dispensing (Q10_10)	○	○	○
Take prescription order from physician over the telephone (Q10_11)	○	○	○
Transfer a prescription to another pharmacy (Q10_12)	○	○	○
Administer a vaccination to a patient (Q10_13)	○	○	○
Draw a blood sample from a patient (finger stick) (Q10_14)	○	○	○
Collect patient medication history from patient (Q10_15)	○	○	○
Call patient prior to medication synchronization to clarify medications to be filled (Q10_16)	○	○	○
Conduct medication reconciliation after patient is discharged from hospital (Q10_17)	○	○	○
Prepare vaccination for administration by pharmacist (Q10_18)	○	○	○
Document pharmacy care in patient records (Q10_19)	○	○	○
Call prescriber for clarification of electronic or written prescription order (Q10_20)	○	○	○

Obtain patient vital signs (blood pressure, heart rate, temperature) (Q10_21)	○	○	○

End of Block: Block4

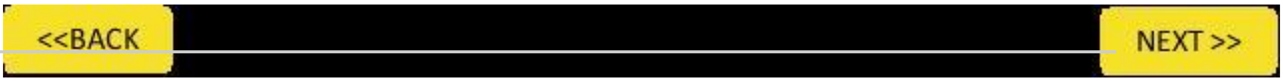

Start of Block: Block5

Q11 Please rate your willingness to perform each of the following emerging tasks as a community pharmacy technician.

	Unwilling (1)	Slightly Willing (2)	Moderately Willing (3)	Very Willing (4)
Perform final prescription verification during dispensing (Q11_1)	○	○	○	○
Take prescription order from physician over the telephone (Q11_2)	○	○	○	○
Transfer a prescription to another pharmacy (Q11_3)	○	○	○	○
Administer a vaccination to a patient (Q11_4)	○	○	○	○
Draw a blood sample from a patient (finger stick) (Q11_5)	○	○	○	○
Collect patient medication history from patient (Q11_6)	○	○	○	○
Call patient prior to medication synchronization to clarify medications to be filled (Q11_7)	○	○	○	○
Conduct medication reconciliation after patient is discharged from hospital (Q11_8)	○	○	○	○
Prepare vaccination for administration by pharmacist (Q11_9)	○	○	○	○
Document pharmacy care in patient records (Q11_10)	○	○	○	○
Call prescriber for clarification of electronic or written prescription order (Q11_11)	○	○	○	○
Obtain patient vital signs (blood pressure, heart rate, temperature) (Q11_12)	○	○	○	○

End of Block: Block5

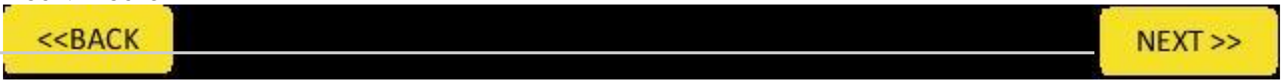

Start of Block: Block6

Q12 Please rate your willingness to complete additional training and/or additional certifications in order to perform these more advanced emerging tasks.

	Unwilling (1)	Slightly Willing (2)	Moderately Willing (3)	Very Willing (4)
Perform final prescription verification during dispensing (Q12_1)	○	○	○	○
Take prescription order from physician over the telephone (Q12_2)	○	○	○	○
Transfer a prescription to another pharmacy (Q12_3)	○	○	○	○
Administer a vaccination to a patient (Q12_4)	○	○	○	○
Draw a blood sample from a patient (finger stick) (Q12_5)	○	○	○	○
Collect patient medication history from patient (Q12_6)	○	○	○	○

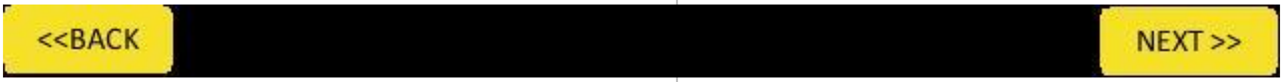

Call patient prior to medication synchronization to clarify medications to be filled (Q12_7)	○	○	○	○
Conduct medication reconciliation after patient is discharged from hospital (Q12_8)	○	○	○	○
Prepare vaccination for administration by pharmacist (Q12_9)	○	○	○	○
Document pharmacy care in patient records (Q12_10)	○	○	○	○
Call prescriber for clarification of electronic or written prescription order (Q12_11)	○	○	○	○
Obtain patient vital signs (blood pressure, heart rate, temperature) (Q12_12)	○	○	○	○

End of Block: Block6

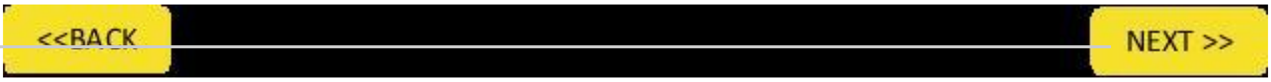

Start of Block: Block7

Q13 Please rate your level of agreement with each of the following statements in regard to pharmacy technicians working in your pharmacy.

	Strongly disagree (1)	Disagree (2)	Somewhat disagree (3)	Neither agree nor disagree (4)	Somewhat agree (5)	Agree (6)	Strongly agree (7)
When implementing a new service, technicians at my workplace may receive training to perform the tasks. (Q13_1)	○	○	○	○	○	○	○
Current staffing levels at my pharmacy may not support technicians assuming additional tasks. (Q13_2)	○	○	○	○	○	○	○
At my workplace the technicians work well with the pharmacists. (Q13_3)	○	○	○	○	○	○	○
I would be excited to have a new opportunity if we implemented a new service at my pharmacy. (Q13_4)	○	○	○	○	○	○	○
Performing new tasks in my workplace helps keep my work interesting. (Q13_5)	○	○	○	○	○	○	○
I don't usually feel stress when working at my job. (Q13_6)	○	○	○	○	○	○	○
If I had to learn to do new tasks for my job, I feel confident that I could do so readily. (Q13_7)	○	○	○	○	○	○	○
My employer classifies technicians based on specialized skills. (Q13_8)	○	○	○	○	○	○	○

My fellow pharmacy staff would be supportive of implementing a new service in the pharmacy. (Q13_9)	o	o	o	o	o	o	o
At my workplace the managers would be supportive when technicians are performing new tasks. (Q13_10)	o	o	o	o	o	o	o
There is enough time in my current workday to complete additional tasks and responsibilities. (Q13_11)	o	o	o	o	o	o	o

End of Block: Block7

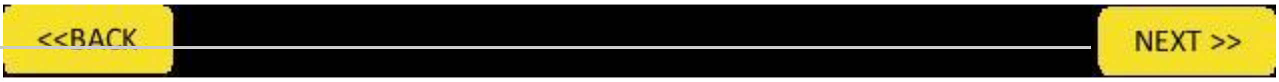

Start of Block: Block8-Demographics_CE

Q14CE What is your gender identity?

- o Male (1)
- o Female (2)
- o Other (0)

Q15CE What is your age?

_______ years (1)

Q16CE Please provide any comments you have about emerging tasks for pharmacy technicians or this survey.

Essay sized text entry

QEnd/CE You have reached the end of the survey. Thank you for taking time to contribute.

You may use the << BACK and NEXT >> buttons to review your answers. If you are satisfied with your answers, please click the SUBMIT button to record your responses.

End of Block: Block8-Demographics_CE

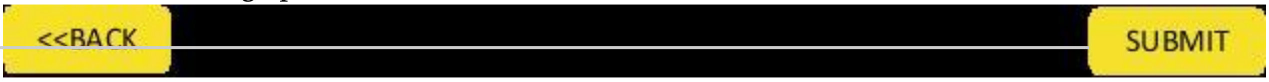

Thank you message returned

Thank you for your time spent taking the survey and thank you for your service to the pharmacy profession.

SCREENED-OUT

Start of Block: Block9-End/Screened-Out

Q2-S How many years did you work as a pharmacy technician?

_______ years (1)

Q3-S What is your gender identity?

- o Male (1)
- o Female (2)
- o Other (0)

Q4-S What is your age?

_______ years (1)

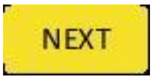

End of Block: Block9-End/Screened-Out

Thank You message returned for Screened-Out

Thank you for your time spent taking the survey and thank you for your service to the pharmacy profession.
